# Effects of Rare Earth Metals on Steel Microstructures

**DOI:** 10.3390/ma9060417

**Published:** 2016-05-27

**Authors:** Fei Pan, Jian Zhang, Hao-Long Chen, Yen-Hsun Su, Chia-Liang Kuo, Yen-Hao Su, Shin-Hau Chen, Kuan-Ju Lin, Ping-Hung Hsieh, Weng-Sing Hwang

**Affiliations:** 1 Department of Materials Science and Engineering, National Cheng Kung University, Tainan 70101, Taiwan; xiaojian529@gmail.com (J.Z.); yhsu@mail.ncku.edu.tw (Y.-H.S.); jdempire1981@gmail.com (C.-L.K.); den702@hotmail.com.tw (S.-H.C.); hann0613@gmail.com (P.-H.H.); 2 Chengdu Base, Panzhihua Iron & Steel Research Institute Co., Ltd., Chengdu 610399, China; 3 Electronic Engineering, Kao Yuan University, Kaohsiung 82151, Taiwan; t11033@cc.kyu.edu.tw; 4 Steelmaking Process Development Section, China Steel Corporation, Kaohsiung 81233, Taiwan; 150151@mail.csc.com.tw (Y.-H.S.); t120@mail.csc.com.tw (K.-J.L.)

**Keywords:** rare earth metals, steel, microstructure

## Abstract

Rare earth metals are used in semiconductors, solar cells and catalysts. This review focuses on the background of oxide metallurgy technologies, the chemical and physical properties of rare earth (RE) metals, the background of oxide metallurgy, the functions of RE metals in steelmaking, and the influences of RE metals on steel microstructures. Future prospects for RE metal applications in steelmaking are also presented.

## 1. Introduction

Rare earth (RE) metals, most of which are located in China, are widely used in steel metallurgy because of their remarkable properties, such the ability to induce a refined microstructure and modify inclusions [1]. RE metals have thus been added into steel to meet specific requirements [2], and they play an important role in improving the quality of the resulting material. Technologies related to the addition of RE metals into steels are thus being used to develop the next generation of steels.

In the early 1990s, the only industrial application need of metal alloys was to improve strength at room temperature under static loading [1]. However, metallurgical researchers were only able to improve strength at the expense of toughness [3]. To meet the needs of the automotive industry, the development of next-generation steel aims at improving both toughness and strength. A high degree of toughness is a fundamental property for structural materials, but paradoxically, the strength of such material is weakened when the toughness increases.

RE elements have very strong chemical activity because of their unique electronic structures, where the valence state of the 4f channel is variable [4]. RE elements can be used to reduce the amount of oxygen and sulfur to a magnitude of 10^−6^, and RE metals can dissolve in iron to form an alloy solution. The dissolved rare earths exist and function in steel, acting as defects based on their difference from iron atoms. Such properties mean that RE metals can be used as purifying agents and inclusion modificators, making them promising elements to promote the strength and toughness of next-generation steel. The history [4] of maraging steel in 20th century shows that only one factory successfully produced this material by using RE lanthanum, and thus there is still great potential to improve the properties of steel by using this technology.

Due to environmental concerns, the large energy consumption associated with producing steel, and the great demand for special steels with better performance, it is necessary to develop advanced technologies for steel manufacturing, in order to improve the material properties, reduce the cost and cut pollution. In this context, oxide metallurgy, especially technologies concerning RE-metal-modified steel, can be used to create advanced steel with high toughness and strength at lower cost and energy consumption.

## 2. Research Background of Oxide Metallurgy

Steel has many material applications due to its strength, remarkable plasticity, and excellent toughness [5]. However, the development of next-generation steel and steel processing must consider environmental damage and resource shortages. Since the 1990s, a rising number of institutes in Japan [6], mainland China [7], South Korea [8], America [9], and the European Union [9] have started large scientific research projects aimed at developing steel with great strength and a long lifetime. The fundamental theories and processing techniques of ultra-fine grains and texture in steel are needed in these efforts, in order to better study the force properties and characteristics of steel.

Improving the properties of steel, which are influenced by the chemical content and inclusions, is the main method used to realize next-generation steels. From the 1960s to the 1970s, fine textures were gained due to the thermal processing techniques used in the first and second generations of such advanced steels by controlling both rolling and the cooling rate after rolling. The steel properties were thus improved dramatically by utilizing the effects of micro-alloy elements on re-graining, phase transformation, and grain growth [10,11].

**Figure 1:**
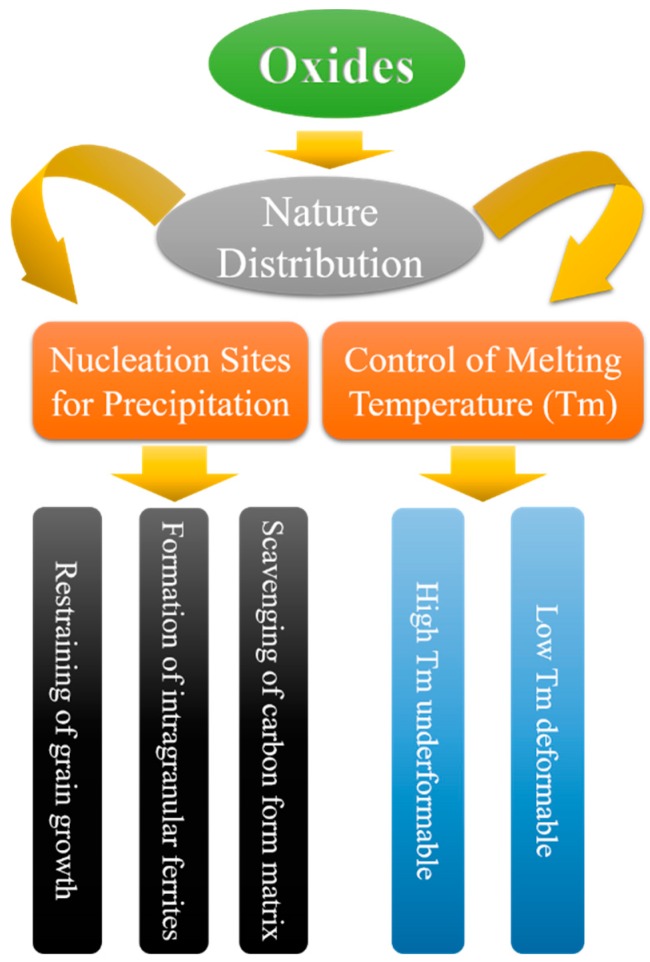
Concept of oxide metallurgy [12].

Non-metallic inclusions (oxides, sulfides, and nitrides) in steel damage the continuity of steel substrates and affect the material properties. The quality of steel is influenced by inclusions with different geometries, force conditions, chemical conditions, and physical conditions, and these would affect the hardness and melting point of the resulting material. Traditionally, inclusions with sizes of less than 1 μm were thought to make little difference to the surface defects and strength of steel. In the 1970s, researchers found that inclusions with sizes of around 1 μm induce intra-granular ferrite (IGF), and thus the steel texture could be refined and the strength and toughness could be significantly improved in the heat affected zone (HAZ). This phenomenon has drawn a lot of attention, because inclusions with sizes of around 1 μm, which can form during solidification and cooling, are very difficult to remove. Indeed, many studies of oxide metallurgy still utilize such inclusions during soldering and metal smelting. Oxide metallurgy, which can refine grains without pressure processing, has drawn much research attention, especially for slab steel and structural steel, for which grain refinement cannot be realized via pressure processing. The concept of oxide metallurgy was first developed up by researchers at Nippon Steel Company in 1990, although it was already known that improvements in grain refinement and soldering properties are related to inclusion size distribution. The concept of oxide metallurgy is shown in Figure 1, and explained below.

- Controlling the oxide distribution and properties in steel (chemical content, melting point, size, and size distribution).
- Utilizing oxides as the core for heterogeneous nucleation to refine grains and, at the same time, as the core for heterogeneous nucleation of sulfides, nitrides, and carbides to control the segregation distribution of sulfur, nitrogen, and carbon, respectively.
- Suppressing grain growth by pinning the austenitic grain boundary at high temperature with the help of oxides, sulfides, nitrides, and carbide; utilizing the inclusions dissolved in the austenite to affect the transformation from austenite to ferrite and induce intra-grain ferrite; improving the processing properties of steel by forming carbide in the steel substrate.

**Figure 2:**
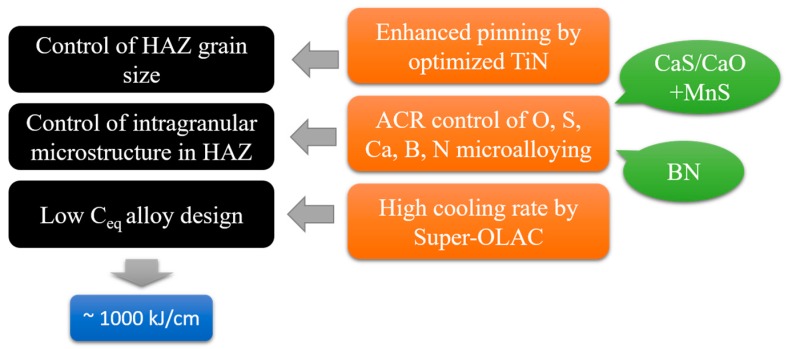
Characteristics of JFE EWEL [26]. OLAC: online accelerated cooling; ACR: atomic concentration ratio.

**Figure 3:**
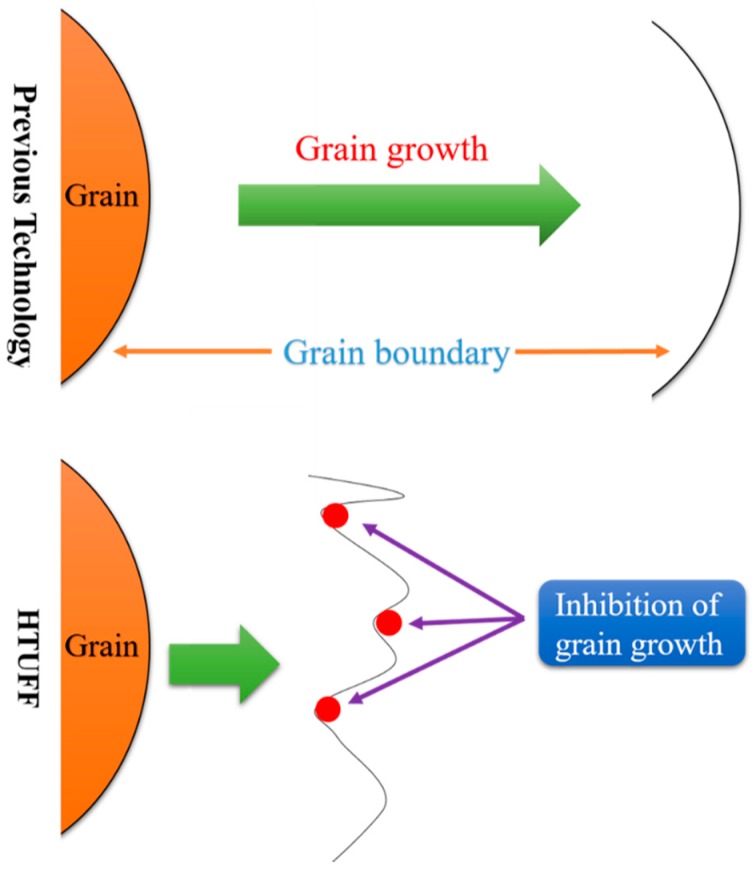
Characteristics of HTUFE [27].

**Figure 4:**
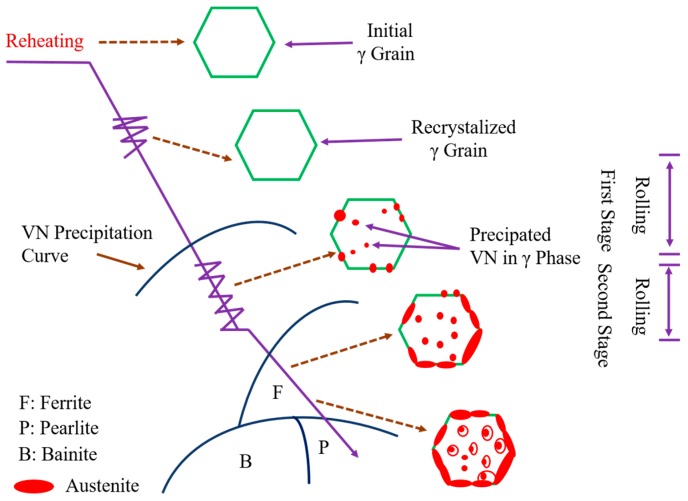
Third-generation TMCP principles for texture refinement [28].

Successful industrial applications of oxide metallurgy include JFE EWEL [13] (Excellent Quality in Large Heat Input Welded Joins), produced by JFE Steel Corporation, Japan, and the HTUFF [14] (Super High HAZ Toughness Technology with Fine Microstructure Imparted by Fine Particles) technique, developed by Nippon Steel. The characteristics of these two techniques are shown in Figure 2 and Figure 3. Both techniques improve steel properties by using existing inclusions without changing the chemical content and refining material textures without processing. Recently, a third-generation thermo-mechanical control process (TMCP) technique [15,16] (TMCP-Oxide metallurgy) was developed, as shown in Figure 4.

The main mechanism of oxide metallurgy involves (a) texture refinement and toughness improvement realized via intra-granular ferrite (IGF), which is induced by dispersing small inclusions in *γ* grains by heterogeneous nucleation; and (b) texture refinement after phase transformation, as realized by pinning grain boundaries where tiny inclusions precipitate.

The metallurgical phenomena related to oxides are crystallization, segregation, grain growth, and phase transformation during deoxidization, casting, rolling, hot and cold molding, and thermal processing. All these phenomena are related to heterogeneous nucleation in steel. Techniques and skills that are closely related to oxide metallurgy are controlling the type of oxide used and the choice of oxidizer [13], controlling the inclusion size and the needed amount of inclusions to induce nucleation for IGF [14], controlling the grain size of austenite [15,16], exploring effects of oxygen concentration on inclusion size and amount [17], studying the influence of cooling rate and nucleation temperature on IGF nucleation [18,19] and exploring the mechanism of IGF induced by inclusions [14,20,21,22,23]. The addition of rare earth (RE) metals in steel can not only form rare earth oxides, but also rare earth sulfides and nitrides [24,25]. The rapid and complete desulfurization that occurs in this process can lower the amount of sulfur to a very low level, and the rare earth sulfides that form can modify the compositions and shapes of the sulfide inclusions, thus improving inclusion plasticity during hot working, as well as the steel’s mechanical properties [24]. The rare earth nitrides which form when adding RE metals in steel improve not only the mechanical properties but also the erosion properties of the steel. The diffusion of RE metals in steel can produce finer nitrides, better microstructures and higher microhardness, which are needed to improve its erosion resistance [25].

## 3. Function of Rare Earth Metals in Steel

The chemical properties and states of RE metals have been known for a long time, but their applications were not studied extensively. During World War II, it was found that the addition of RE metals to steel could significantly improve its properties, since then, RE metals have become widely applied in steel manufacturing [29,30].

### 3.1. Brief Introduction to Rare Earth Elements

RE elements are known as lanthanide elements. The International Union of Pure and Applied Chemistry (IUPAC) defines RE metals as the 15 lanthanide elements (La, Ce, Pr, Nd, Pm, Sm, Eu, Gd, Tb, Dy, Ho, Er, Tm, Yb, and Lu), with atomic numbers ranging from 57 to 71, and two elements (Sc and Y) closely related to these lanthanide elements. RE elements, represented as [Xe]4f^n^6s^2^ or [Xe]4f^n−1^5d^1^6s^2^, have similar electronic shell structures, where electron numbers range from 0 to 14 in the 4f transitional level, and the outer electrons consist of one electron in the d channel and two electrons in the s channel. RE metals have atomic diameters ranging from 1.641 to 2.042 Å, and are slightly more electronegative than alkali earth metals, and so can lose electrons and easily become positive ions. RE metals are thus chemically active in liquid steel. RE metals also react with sulfur and oxygen to form products with high melting points, and can thus reduce the harm to the properties of steel due to impurities via reactions with plumbum and stannum [31].

Cerium (Ce) is positively trivalent and is the second lanthanide element belonging to the light RE metals located in Group IIIB, the sixth period of the periodic table. The electronic structure of cerium makes it a strong reducing agent. At room temperature, cerium can react with oxygen and under some conditions it can react with non-metallic elements [32]. Table 1 [32] shows its physical properties.

### 3.2. Function of Cerium in Steel

Cerium is widely applied in steel, in processes that can be classified into purification modification of inclusions and micro-alloys [33,34]. Cerium has the ability to improve the cleanliness of steel, as, for example, it can deoxidize and desulfurize steel and prevent harm due to hydrogen, phosphor, arsenic, stannum and lead [1]. Cerium can not only purify liquid iron but also refine ingots and the microstructures of continuous casted steel. This is because cerium can modify the properties, distribution and shapes of inclusions [35]. The dissolving of cerium in the crystal lattice of iron results in lattice distortion that improves the toughness of the resulting steel [36]. Cerium can also segregate on the grain boundaries and thus overcome the weakness due to the presence of other elements [37,38].

### 3.3. Purification of Steel by Using Rare Earth Metals

The purification (deoxidization, desulfurization, and removal of elements with low melting points) of steel by RE metals relies on their reactions with oxygen, sulfur, lead, arsenic, tin, and antimony, which can easily form non-metallic compounds with high melting temperatures. Purification is achieved when these non-metallic compounds float to the upper slag and thus amount of impurities in the resulting steel can be reduced. Based on the Gibbs free energy of RE compounds, when the oxygen content is sufficiently low, RE elements combine with sulfur first and then remove it [15,16].

### 3.4. Modification of Inclusions

RE elements can affect the structure, morphology, and distribution of inclusions and impurities, thus eliminating defects in steel. The properties of steel are greatly improved when its grains are refined by RE elements, and the products from deoxidization and desulfurization are modified by the addition of RE elements to liquid steel. Products with high melting points easily cluster and float, improving the inclusion distribution. Inclusions with high melting temperatures are randomly distributed around the grain boundary when a small amount of RE metal is added [39]. Complete desulfurization can be achieved if the ratio of [RE] to [S] is precisely controlled. Modification can be achieved when the [RE]/[S] ratio is >3 [40]. Compounds of RE elements and sulfur can replace manganese sulfide (MnS), fully eliminating elongated manganese sulfide inclusions. RE compounds, which look like small spheres or spindles evenly distributed in steel, do not deform during casting. Because RE inclusions and steel have similar thermal expansion coefficients, the fatigue strength of steel is significantly improved with the addition of RE, because stress concentrations are avoided during casting [41].

### 3.5. Micro-Alloying

In micro-alloying, the microstructure and texture are influenced by solid dissolution and the reaction of the solid phase, and thus these can be manipulated to improve the properties of steel. The criteria for judging a micro-alloy are based on the element state, dissolution, and amount dissolved in steel. For metallic materials, solid dissolution means the atoms exist in substrate matrices and there are random defects. For RE metals, minute quantities dissolve in steel rather than form a solid dissolution according to the Hume-Rothery principle, and the atomic diameters of RE metals are 0.5 times larger than those of iron atoms. The diameters of RE metals are altered by polarization between metallic and non-metallic atoms. For example, when the degree of ionization of a lanthanum atom reaches 60%, the atomic covalent diameter of lanthanum is reduced to 0.1277 nm from 0.1877 nm, which is 5.5% times larger than that of an iron atom (0.1210 nm). RE atoms form a substitutional solid solution in the crystal by occupying the lattice section points using a vacancy diffusion mechanism. The tested solubility of RE metals in steel is around 10^−6^~10^−5^ ppm magnitude based on the electrolysis of RE inclusions and plasma mass spectrometry, using physical and chemical methods and internal friction peaks. Tiny amounts of an RE metal dissolved in steel can distort the iron crystal lattice and enhance the strength of the steel. RE metals tend to segregate at grain boundaries and eliminate the local weaknesses due to sulfur and phosphor atoms in steel, improving the strength of grain boundaries and shock resistance [36,42,43,44].

### 3.6. Grain Refinement

Solid particles of RE compounds act as heterogeneous nucleation sites and can segregate at the interface of crystalline structures, hindering cell growth; thermodynamic conditions are thus needed to refine the steel grains with the addition of RE metals [2]. The effects of adding RE metals at various amounts on macroscopic and microscopic crystal structures have been investigated. One study found that the distance between the crystalline structures decreased significantly and that the solidification of an interdendritic liquid film with low melting temperature was promoted with the addition of RE metals [45]. The functions of RE metals in high-sulfur steel, in which RE compounds act as the core of non-spontaneous crystallization, are grain refinement and the promotion of the equiaxed grain rate. The effects of RE metals on the crystal structure of low-sulfur steel are reflected in the thinning space of the dendrite arms. Research also shows that the heterogeneous nucleation sites mainly composed of Ce_2_O_3_, which are formed after the addition of RE metals in liquid steel due to their high melting point, can have the effects on grain size of ultra-low carbon steel. The yield strength of the material was significantly improved and the cast grain size was significantly reduced due to the increasing number of nucleation sites of the solid and liquid phases [46].

## 4. Influence of Rare Earth Metals and Cerium on the Microstructures of Steel

In carbon RE steel, RE atoms exist in cementite as replacements of iron atoms rather than as carbides. RE atoms tend to segregate at the interface of ferrite and cementite due to their large radius and high aberration energy. RE atoms are thus mainly distributed at the interface of cementite alloys and grain boundaries. The grain sizes of austenite decrease significantly with the addition of a greater amount of RE metals. The austenite grain size can be controlled to around 10 μm when the amount of RE metals is more than 50 ppm. However, the austenite grain size does not change significantly with further increases in the amount of RE [47].

Grain boundaries are the preferred nucleation sites for the precipitated phase. The existence of dissolved atoms and the higher rate of atomic diffusion at grain boundaries than seen with volume diffusion or lattice diffusion contribute to nucleation and grain growth for the precipitated phase. The segregation, diffusion, and precipitation of RE atoms at grain boundaries can greatly affect steel properties. A limited amount of RE metals can improve the preservative ability of steel, while an excess amount of RE metals can deteriorate this. It has been reported that steel with 21 ppm of RE metals has the optimal properties of hardness and inclusion modification [48].

### 4.1. Effects of Rare Earth Metals on Microstructure of Cast 0.4C–5Cr–1.2Mo–1.0V Steel

**Figure 5:**
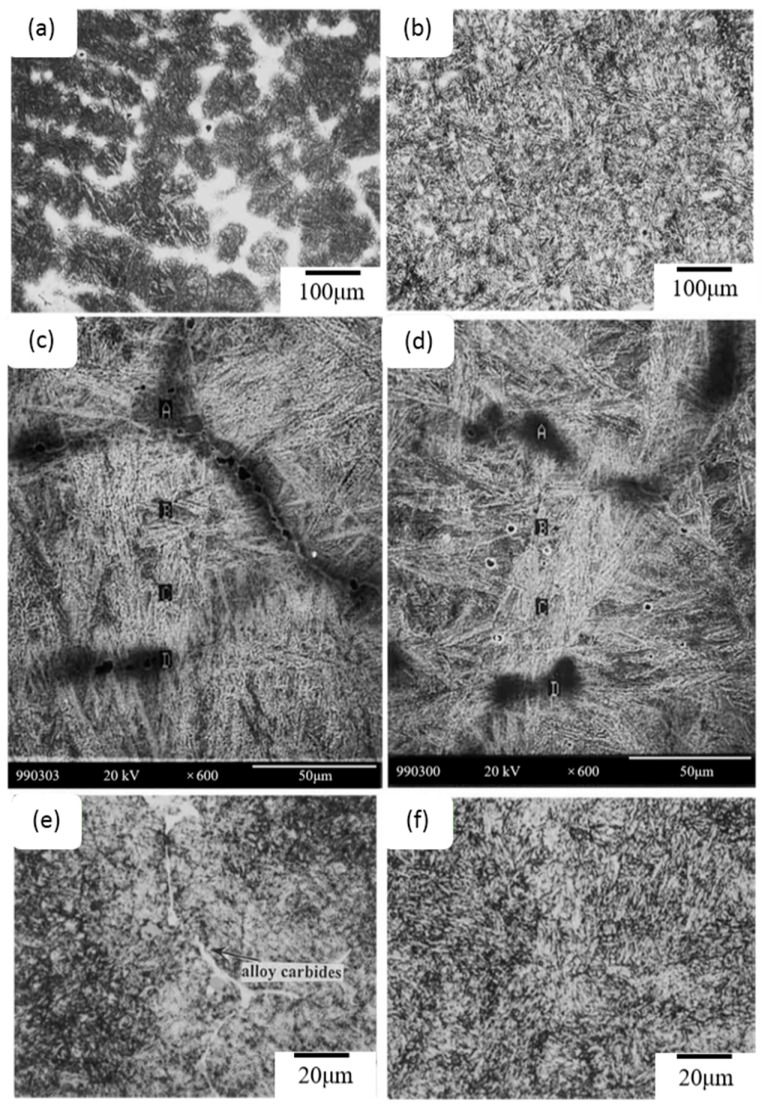
Cast microstructures of (**a**) CH13 steel and (**b**) MCH13 steel. Interdendrite areas of (**c**) CH13 steel and (**d**) MCH13 steel. Tempered microstructures of (**e**) CH13 steel and (**f**) MCH13 steel [49].

It has been reported that MCH13 [49], which is produced by the addition of RE metals to CH13 steel, has a more homogeneous cast structure. The cast microstructures of CH13 and MCH13 are shown in Figure 5. The light area is high-carbon martensite with austenite and carbide, and the dark area is low-carbon martensite. Comparing Figure 5a,b, the light area in CH13 has a net-like distribution dispersion, indicating a dendrite structure, but the light area in MCH13 has a dot-like distribution dispersion. Quantitative analysis shows that adding RE metals to steel greatly decreases the number of dendrite structures.

To evaluate the degree of segregation, the segregation ratio *δ* is introduced (*δ_i_ = C_i_*_max_/*C_i_*_min_, where *C_i_*_max_ and *C_i max_* are the maximum and minimum concentrations of element *i* in the interdendritic area, respectively). A study of the segregation ratio *δ* of the main alloying elements in CH13 and MCH13 indicates that the microstructure of MCH13 is more homogeneous.

Many carbides form at the grain boundaries in CH13 steel because of the high concentration of alloying elements, especially at these boundaries and with a low cooling rate of annealing. The formed carbides cannot avoid elimination during austenitization at high temperature. The number of carbides at grain boundaries in MCH13 is much lower due to its more homogeneous cast structure.

**Figure 6:**
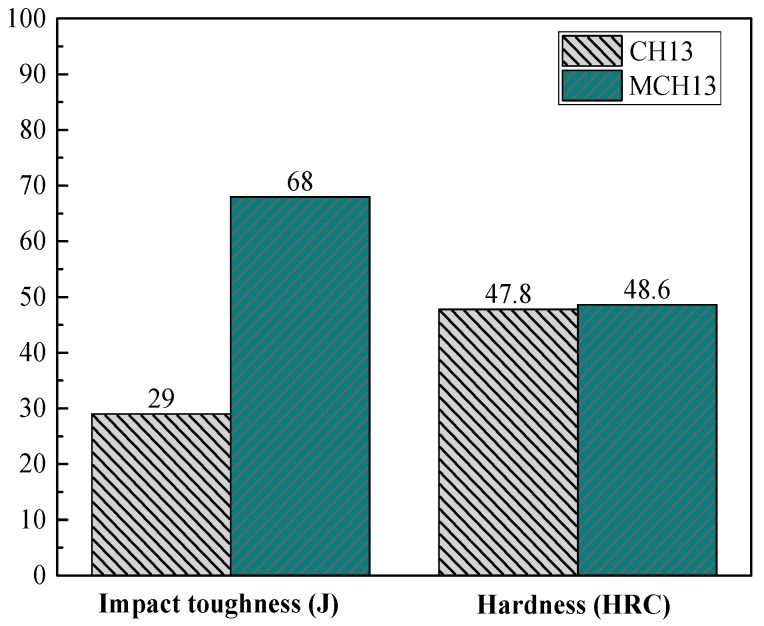
Impact toughness of tempered CH13 and MCH13 steels [49].

As shown in Figure 6, the impact toughness of MCH13 is 39 J larger than that of CH13 for a given hardness. Near fractures of MCH13, many secondary fractures form as the main crack propagates, releasing large quantities of stored elastic energy. However, the longitudinal section of CH13 is much smoother and the secondary fractures are much fewer near the first fracture. The carbides at grain boundaries in CH13 form a net-like structure, and act as obstacles for partial dislocation. This results in a large stress concentration at grain boundaries, leading to tunnel-like void formation. As such, cracks form easily at grain boundaries. Most importantly, the carbide network is eliminated by RE metal addition in MCH13.

Micro-fractures are greatly affected by sulfides and oxides, which can act as quasi-cleavage fracture nuclei. The oxides, which are more effective sites for stress concentration, are larger and longer than the sulfides. The inclusions found in CH13 steel are mostly elongated, but those observed in MCH13 are mostly spherical. Therefore, stress concentration, which results in fracture nuclei, occurs more easily in CH13 steel compared with MCH13 steel.

### 4.2. Effects of Rare Earth Metal and Titanium Addition on the Microstructures of Low-Carbon Fe–B Cast Steel

The addition of RE metal to Fe-B cast steel improves the microstructural spheroidization of the eutectic borides during heating [20]. The addition of RE metal accelerates the diffusion of B and Fe atoms due to the increased number of defects and matrix aberrance near RE atoms, which arise because of their larger radius. When RE metals enter borides, lattice distortion occurs, resulting in an increase in lattice distortion energy and a decrease in boride stability. Therefore, the addition of RE metals and titanium to Fe-B cast steel leads to solidification structure refinement and increases the chance of necking and network breaking. A study of Fe–1.72Cr–2.54C (wt %) samples found that the dissolution of carbides occurs everywhere when RE metals are added. Afterwards, the networks of carbides continue to break up and become thinner and smaller.

The addition of RE metals and titanium to Fe-B cast steel refines austenite, inhibiting the coursing of borides. There is a theory that inoculating agents are most effective when the mismatch is less than 6% [20]. When the mismatch is 6% or 12%, the agents are intermediately effective; the agents are ineffective when the mismatch is larger than 12%. RE oxides, RE sulfides, and compounds of carbon and nitrogen have very high melting points, and their mismatch is much lower than 6%. Therefore, RE oxides and titanium nitride are very effective heterogeneous nucleation sites and can refine the microstructure of Fe-B cast steel.

### 4.3. Effects of Rare Earth Elements on Microstructures in TIG Weldments of AISI 316L Stainless Steel

**Figure 7:**
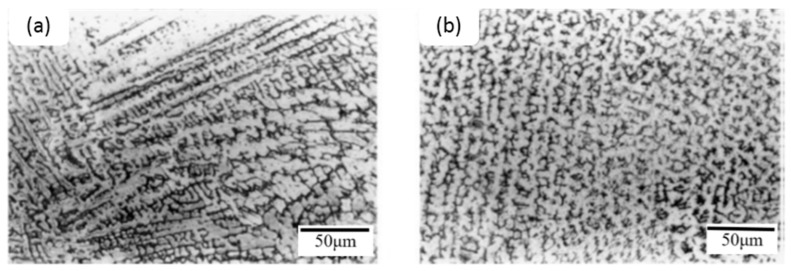
Optical microstructure of 316L TIG welded zone with 0.03% Ce. (**a**) Near fusion boundary and (**b**) weld center (400×) [50].

**Figure 8:**
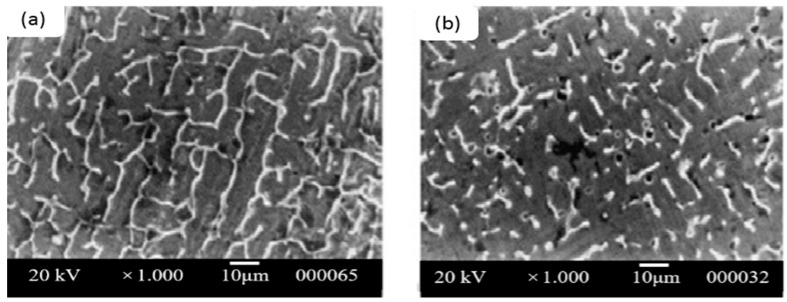
SEM micrographs of weld metal zone with 0.08% Ce + 0.8% Nb. (**a**) Near fusion boundary and (**b**) weld center [50].

It has been reported that the duplex structure of the 316L TIG welded zone [50] is fully vermicular, and directionality was observed near the fusion boundary rather than the center of the TIG weld due to the variations in solidification and growth velocity at fusion boundaries. There are distinct microstructural variations with the addition of cerium, niobium, and their combination in TIG weld zones. Lacy and vermicular ferrite can be seen near the fusion boundary in Figure 7a, and a small amount of vermicular ferrite can be seen near the weld center in Figure 7b. The ferrite number decreases as the amount of cerium in steel increases. Oxides, oxy-sulfides, or sulfides form in the presence of RE metals based on the element activity in the weld pool. RE metal inclusions provide enough driving force when nucleation and inoculation occur during weld pool solidification. RE metal inclusions could lead to fragmentation or dissociation, suppressing columnar growth. Figure 7 and Figure 8 show that the refinement of dendritic structures and minimization of the microsegregation of the interdendritic region are due to the segregation of RE metals into the liquid phase during solidification.

**Figure 9:**
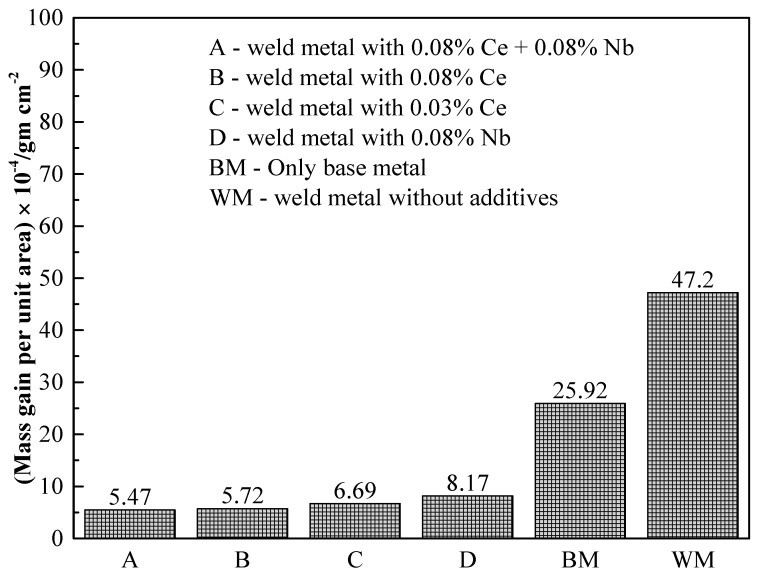
Oxidation behavior of weld zones under various conditions for base metal of 316L stainless steel oxidized at 973 K in PO_2_ = 21.27 kPa for 240 h [50].

Figure 9 shows the improvement in oxidation resistance obtained with various additives. The most effective improvement was obtained with a combination of cerium and niobium. The improvement obtained with cerium in the weld metal zone is better when the same amount of cerium and niobium are added into the weld metal. The excellent oxidation resistance obtained with a combination of cerium and niobium is attributed to the RE metals inhibiting the formation of chromium carbide, thus preventing Cr depletion in steel. Niobium has greater affinity to carbon than it does to oxygen and chromium. Therefore, niobium tends to pick up carbon during welding, even though the cerium plus niobium was wrapped in 316L stainless steel foil with 0.55% carbon.

The tendency for the simultaneous formation of complex compounds during heating is another possible reason for the lower oxidation rate shown in Figure 9. During heating, complex compounds such as (Fe, Cr)_2_O_3_, FeCr_2_O_4_, CeFeO_3_, and NiCr_2_O_4_ may form. However, the reactive element cerium and its oxides are the main reasons for initial grain refinement, because the embedded cerium oxides, which act as a graded seal to accommodate thermal stresses, are more resistive to ionic transport. The oxidation rate thus decreased.

The segregation of Cr, Mo, and V decreases the microstructure via the homogenization of the modified steel sample at 950 °C, 1000 °C, and 1050 °C for 1 h. It has been reported that niobium carbides remain the morphology when homogenized, because of their good stability and low dissolution into austenite [51]. Molybdenum and vanadium carbides segregate at grain boundaries; although these segregated carbides can be reduced by increasing the homogenization temperature, since they dissolve easily into austenite at high temperature. Homogenization at high temperature increases the grain size of austenite while increasing the homogeneity of the microstructure. The dissolution of VC and (Mo,Fe)_6_C particles into austenite results in austenite grain growth with increasing heating temperature and time. The addition of Nb to H13 steel increases steel hardness compared to that of the original steel due to the presence of NbC.

### 4.4. Effects of Rare Earth Element Yttrium on Microstructures of 21Cr–11Ni Austenitic Heat-Resistant Stainless Steel

**Figure 10:**
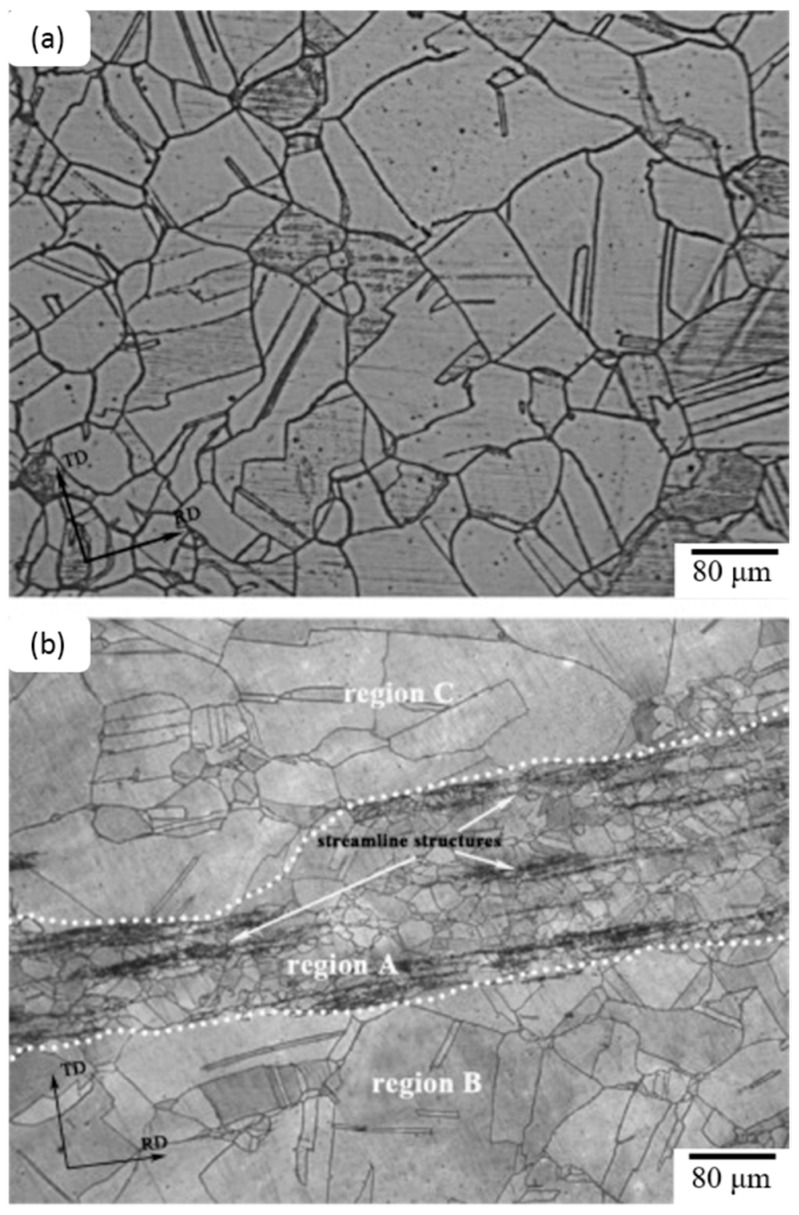
Initial microstructure of test steels in hot-rolled + annealed condition. Steel (**a**) without and (**b**) with RE metals [58].

**Figure 11:**
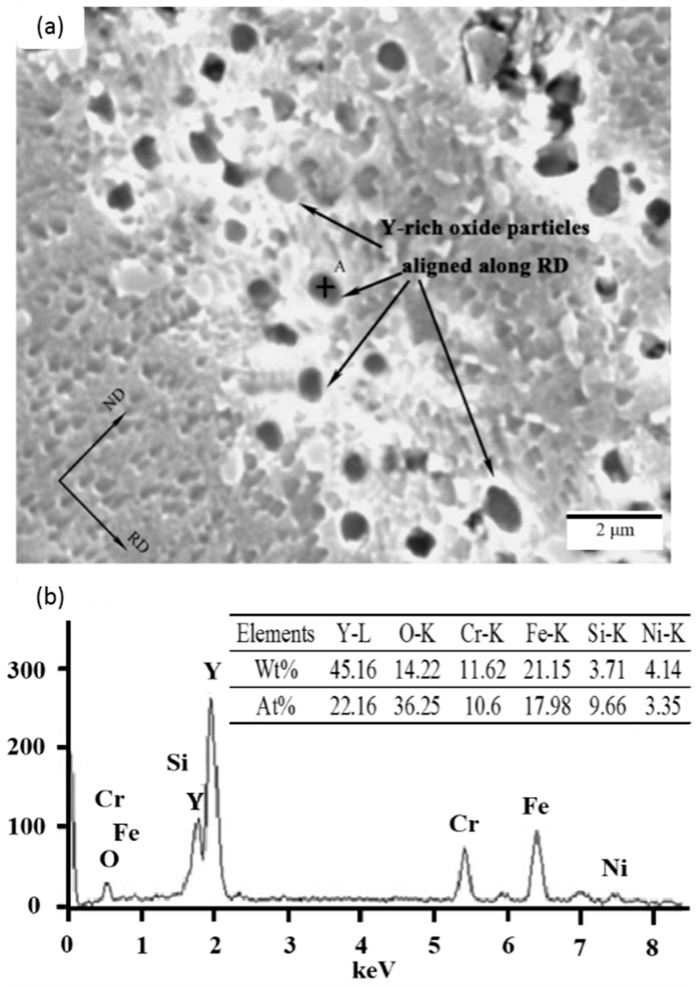
(**a**) SEM image of fine spherical particles aligned along the rolling direction of steel with RE metals, and (**b**) corresponding EDS for spherical particles (point A indicates a particle) [58].

Figure 10a,b shows the microstructures of the initial steel without RE metals and steel with 480 ppm yttrium addition, respectively [52]. Figure 10a shows equiaxed austenitic grains with an average size of 127 μm and lamella-like straight annealing twins. In Figure 10b, streamline structures appear in the austenite matrix. Many spherical inclusions are found in the streamline structures, and are aligned along the rolling direction, as shown in Figure 11a. The size of these aligned inclusions is 300–500 nm. From the results of the energy-dispersive X-ray spectroscopy (EDS) analysis, as shown in Figure 11b, these inclusions are Y-rich. The alignment of these Y-rich inclusions is due to hot rolling [53,54,55].

The average size of grains near Y-rich inclusions is much smaller than that of grains far away. This may be attributed to the microstructure evolution during recrystallization, and grain growth after rolling and annealing. During recrystallization, the Y-rich inclusions stimulate nucleation and thus refine grains. Incoherent arrays of Y-rich spherical inclusions pin grain growth via Zener pinning [56,57], because of the decrease in grain boundaries that intersect with Y-rich particles.

A possible mechanism for grain refinement by Y-rich inclusions is analyzed below. The necessary condition for grain growth was proposed by Rios [59], and this criterion is:

(1) $$F_{gg}>F_{s}+\;F_{p}$$

where *F_gg_* is the driving force of grain growth, *F_s_* is the retarding force of solute atoms, and *F_p_* is the retarding force of inclusions (Zener pinning force). Rios confirmed that solute atoms have little effect on grain growth because of their low movement rate. Therefore, *F_s_* is negligible and the criteria can be re-written as:

(2) $$F_{gg}>F_{p}$$

As such, Equation (2) can be simplified to:

(3) $$F_{gg}/F_{p}>1\;$$

The driving force of grain growth *F_gg_* can be expressed as [60]:

(4) $$F_{gg}=2\gamma \left(\frac{1}{D_{f}\tan\left(\Omega D_{s}/2D_{f}\right)}-\frac{\sqrt{3}}{D_{f}}\right)$$

where *γ* is the grain boundary interfacial energy (assumed to be a constant and independent of grain orientation), and *D_s_* and *D_f_* are the average diameter of normal and abnormal matrix grains, respectively. *Ω* is a parameter that varies within certain limits, depending on *D_s_* and *D_f_*, expressed as [60]:

(5) $$\Omega =\frac{\pi }{2}-\frac{\pi }{6}{\left(\frac{D_{s}}{D_{f}}\right)}^{2}$$

The Zener pinning force introduced by Rois [59] is expressed as:

(6) $$F_{p}=3f\gamma /r$$

where *γ* is the grain boundary interfacial energy, *f* is the particle volume, and *r* is the mean radius of inclusions.

From Equations (4) and (6), the following is derived:

(7) $$\frac{F_{gg}}{F_{p}}=\frac{2r}{3f}\left(\frac{1}{D_{f}\tan\left(\Omega D_{s}/2D_{f}\right)}-\frac{\sqrt{3}}{D_{f}}\right)=\frac{d}{3f}\left(\frac{1}{D_{f}\tan\left(\Omega D_{s}/2D_{f}\right)}-\frac{\sqrt{3}}{D_{f}}\right)$$

where *d* is the diameter of inclusions.

The calculated *F_gg_*/*F_s_* with the reported parameter [61] is around 25, and thus the driving force is much larger than the retarding force. Based on this theory, the grains near Y-rich inclusions should be larger. However, this contradicts the experimental results. Therefore, the stimulation of nucleation during recrystallization should be the main factor of grain refinement.

### 4.5. Effects of Rare Earth Metals on Microstructures of High Speed Steel with High Carbon Content

The eutectic microstructures in the original HCHSS steel are distributed in the form of flakes and networks, where small amounts of coarse block carbides disperse [62]. Most of the microstructures are martensite, austenite, and pearlite. However, the austenite grains and eutectic structures undergo a transformation into fine structures and the lamellar carbides in the eutectic structures become short and fine after the addition of RE metals. With the addition of these, the carbides become distributed in the form of discontinuous networks and grains due to the concentration of RE metal atoms at carbide boundaries [63].

After heat treatment of unmodified steel at temperatures lower than 1000 °C, the change in the microstructure and carbide distribution is slight, and the microstructure remains coarse and a massive distribution of carbides. Carbides start to dissolve into the matrix and become round and smooth when the temperature is increased. The microstructure is finer and the network of additional carbides is disconnected in the modified steel. After heating at 1000 °C, the eutectic carbides not only become isolated and spheroidized, but also transform into a large quantity of granular carbides. The network of carbides disappears and the carbides transform into granules and spheroids after heating at 1050 °C. The eutectic carbides of HCHSS steel transform into disconnected spheroids [64]. The degeneration of eutectic carbides at high temperature is due to carbide dissolution, atomic diffusion, and separation. The carbide network may become disconnected at sharp corners and weak sites, where carbides dissolve faster. The addition of RE metal to steel hinders the formation of eutectic carbide networks and makes eutectic carbides weakly connected, because the RE metal atoms enter the eutectic carbide lattice and distort it [65]. RE metal atoms can then distort the matrix near the lattice and increase the number of vacancies, because their atomic radius is larger than that of iron atoms [66]. RE metal atoms thus play a very important role in dissolving eutectic carbides and spheroidizing carbides.

### 4.6. Effects of Rare Earth Metals on the Microstructures of High-Silicon Cast Steel

The refinement of austenite grains [67] is an efficient way to enhance the toughness of bainitic steel [68]. The variation of grain size in the parent phase is a result of the modification of the grain boundaries [67]. The modification of grain boundaries and atomic diffusivity may affect the kinetics and products of the degeneration of the parent phase.

**Figure 12:**
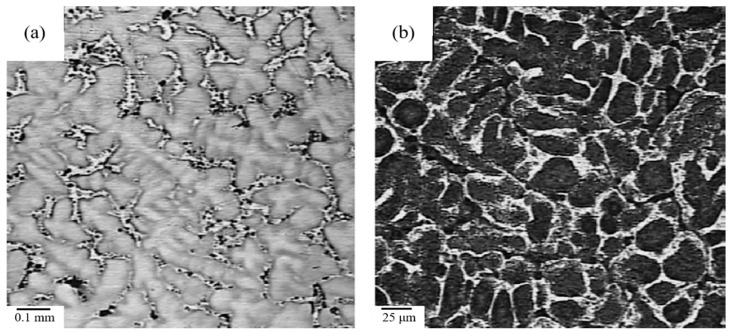
Microstructure of primary austenite grains of AHS steel: (**a**) steel A and (**b**) steel B [67].

Figure 12 shows the austenite grains before and after modification. From the microstructures without modification, it can be seen that the austenite grains are coarse dendrites with long primary axes and short secondary axes. The austenite grains become well refined and equiaxed when titanium, vanadium, and RE elements are added. The compounds of these additives and dissolved carbon, nitrogen, and oxygen have very high melting points. These compounds act as heterogeneous nuclei for austenite formation in the melt. Based on the Turnbull-Vonnegut theory, the lattice disregistry *δ* between nucleation agents and the nucleated phase is key for determining whether the agents serve as heterogeneous nuclei. It has been reported that the lattice disregistry between VC and *γ*-Fe is about 8.38%, and that between Ce_2_O_3_ and austenite is 5.92% less than 6% [69]. Therefore, Ce_2_O_3_ is an effective heterogeneous nucleus.

The dendrite structures are eliminated after modification, and an entirely equiaxed structure appears after austempering for 1 h at 350 °C after austenitization for 1 h at 930 °C. The microstructures of samples after austempering at various temperatures indicate the formation of carbide-free ausferrite with parallel fine beinitic ferritic laths and retained austenite films, rather than the traditional acicular lower bainite and feathered upper beinite. With an increase in austempering temperature, the bainitic ferrite laths widen and carbon-rich austenite thickens without carbide precipitation. The amount of block-shaped retained austenite decreases, the banite ferrite becomes shorter, and the distribution of austenite tends to become more homogeneous. When the volume ratio of thin film retained austenite to block-shaped retained austenite in the bainitic structure is greater than 0.9, the steel may exhibit higher impact toughness at a high strength level than that achieved under the tempered martensitic condition [70,71,72,73,74]. The volume fraction of the retained austenite in the non-modified steel is greater than that in the RE-metal-modified steel at low austempering temperature; however, the fractions of these two steel samples are similar at high austempering temperature. This is attributed to the finer austenite grains and the heterogeneous nuclei of bainitic ferrite, which enable the precipitation of (Ti,V) (C,N) from austenite at low austempering temperature. The very high diffusion rate and the rapid increase in carbon in the residual austenite are the reasons why the volume fractions are similar at a high austempering temperature.

### 4.7. Effects of Rare Earth Metals on the Microstructures of Cast High-Speed Steel Rolls

Eutectic carbides form large quantities of agglomerates of MC + *γ* and metal-stable carbides M_2_C during final solidification without RE metal modification. The uniform distribution of MC carbides can be observed with the addition of RE metals into the high-speed steel. In addition, stable M_6_C + *γ* eutectic undergoes a transformation in the final solidification stage. When RE metals are added to the steel, these atoms combine with carbides and form RE metal carbides, reducing the lattice size of MC carbides andhindering their growth, which leads to a uniform distribution of grains. RE metal atoms promote the formation of M_6_C and long pole-shaped MC carbides, as confirmed by the appearance of tree-like grains [75].

### 4.8. Effect of Rare Earth Metals on Microstructures of 17-4PH Steel

The addition of RE metals to a plasma nitrocarburizing atmosphere can increase the depth of the nitrocarburized layer [76]. RE metal addition also changes the microstructure of the nitrocarburized layer, affecting the mechanical properties of the surface layer. Although the addition of RE metals to the modified layer does not change the surface phases, which are mainly *γ*’-Fe_4_N and *γ*’-Fe and some CrN, it increases the diffraction intensity of *γ*’-Fe_4_N and *γ*’-Fe, indicating increased amounts of these phases [77,78].

## 5. Conclusions and Future Prospects

Magnesium has strong affinity to oxygen and sulfur and can form small inclusions [79]. RE metals have slightly higher electronegativity than those of alkali and alkali earth metals, and can thus easily lose electrons and become positive ions. RE metals react with sulfur and oxygen to form products with high melting points, and can resolve the harm to steel properties from impurities via reactions metals with plumbum and stannum [31]. That is to say, RE metal elements have the ability to deoxidize and desulfurize steel more thoroughly than magnesium, which can reduce the amounts of oxygen and sulfur to very low levels, and the resulting rare earth oxides and sulfides can modify the compositions and shapes of the magnesium oxide and sulfide inclusions [75]. It is indicated that steel modified by RE metals can have a number of better properties *i.e.*, toughness, fatigue resistance, corrosion resistance and hot ductility, and thus have a wider range of applications than steel modified by magnesium in the automobile industry [1]. The addition of RE metals to liquid iron improves the microstructure of steel and induces acicular ferrite, improving the material properties of the metal. The discovery of methods for adding appropriate amounts of RE metals to steel is thus key for the development of next-generation steelmaking technology. In short, adding RE metals is a promising approach for improving the quality of steel.

## Figures and Tables

**Table 1:** Physical properties of cerium [32].

State	Density (G/Cm^3^)	Atomic Diameter (Å)	Electroneg Ativity	Melting Point (°C）	Boiling Point (°C)	Fusion Heat (Kj/Mol)
Gray metal	8.24	1.824	1.12	798	3426	5.46

## References

[1] Wang L. (2004). Application prospects and behavior of RE in new generation high strength steels with superior toughness. J. Chin. Rare Earth Soc..

[2] Zhou D., Peng P., Xu S., Liu J. (2004). Reasearch and application of rare earth in steel. Res. Stud. Foundry Equip..

[3] Hu X., Zi Y. (2004). The application of rare earth in steel. Spec. Steel Technol..

[4] Ji J. (2001). Addition of the rare earth element to steels-an important approach to developing steels in the 21st century. Chin. Rare Earths.

[5] Hwang W., Fu J. (2012). Steel Metallurgy: Secondary Refining of Oxide Metallurgy.

[6] Satoh A. Research project on innovative steels in Japan (STX-21 Project). Proceedings of the Ultra Steel 2000: International Workshop on the Innovative Structural Materials for Infrastructure in 21st Century.

[7] Weng Y. New generation of Iron and steel material in China. Proceedings of the Ultra Steel 2000: International Workshop on the Innovative Structural Materials for Infrastructure in 21st Century.

[8] Lee W. Development of high performance structural steels for 21st century in Korea. Proceedings of the Ultra Steel 2000: International Workshop on the Innovative Structural Materials for Infrastructure in 21st Century.

[9] Salvatori I. In European Project on Ultra Fine Grained Steels by Innovative Deformation Cycles. Proceedings of the Second International Conference on Advanced Structural Steels.

[10] Sekine H., Maruyama T., Kageyama H., Kawashima Y. (1981). Grain refinement through hot rolling and cooling after rolling. Thermom. Process. Microalloyed Austenite.

[11] Weng Y. (2003). Steel with Ultra-Fine Grain: Refinement Theory of Steel Structure and Control Technology.

[12] Ogibayashi S. (1994). Advances in technology of oxide metallurgy. Nippon Steel Tech. Rep..

[13] Sarma D.S., Karasev A., Jönsson P. (2009). On the role of non-metallic inclusions in the nucleation of acicular ferrite in steels. ISIJ Int..

[14] Ricks R., Howell P., Barritte G. (1982). The nature of acicular ferrite in HSLA steel weld metals. J. Mater. Sci..

[15] Lee J. (1994). Evaluation of the nucleation potential of intragranular acicular ferrite in steel weldments. Acta Metall. Mater..

[16] Zhang Z., Farrar R. (1996). Role of non-metallic inclusions in formation of acicular ferrite in low alloy weld metals. Mater. Sci. Technol..

[17] Goto H., Miyazawa K., Yamaguchi K., Ogibayashi S., Tanaka K. (1994). Effect of Cooling Rate on Oxide Precipitation during Solidification of Low Carbon Steels. ISIJ Int..

[18] Goto H., Miyazawa K., Yamada W., Tanaka K. (1995). Effect of cooling rate on composition of oxides precipitated during solidification of steels. ISIJ Int..

[19] Yang Z., Wang F., Wang S., Song B. (2008). Intragranular ferrite formation mechanism and mechanical properties of non-quenched-and-tempered medium carbon steels. Steel Res. Int..

[20] Bramfitt B.L. (1970). The effect of carbide and nitride additions on the heterogeneous nucleation behavior of liquid iron. Metall. Trans..

[21] Byun J., Shim J., Cho Y., Lee D. (2003). Non-metallic inclusion and intragranular nucleation of ferrite in Ti-killed C–Mn steel. Acta Mater..

[22] Grong O., Matlock D.K. (1986). Microstructural development in mild and low-alloy steel weld metals. Int. Met. Rev..

[23] Ohkita S., Horii Y. (1995). Recent Development in Controlling the Microstructure and Properties of Low Alloy Steel Weld Metals. ISIJ Int..

[24] Arunachalam V., Ramachandran S. (1980). Rare Earths in Steel Technology. Sci. Technol. Rare Earth Mater..

[25] Cheng X., Xie C. (2003). Effect of rare earth elements on the erosion resistance of nitrided 40Cr steel. Wear.

[26] Suzuki S., Ichimiya K., Akita T. (2005). High tensile strength steel plates with excellent HAZ toughness for shipbuilding: JFE EWEL technology for excellent quality in HAZ of high heat input welded joints. JFE Tech. Rep..

[27] Kojima A., Kiyose A., Uemori R., Minagawa M., Hoshino M., Nakashima T., Ishida K., Yasui H. (2004). Super high HAZ toughness technology with fine microstructure imparted by fine particles. Shinnittetsu Giho.

[28] Liu Z., Kuwabara M. (2007). Up-to-date progress of technology of oxide metallurgy and its practice. Steelmaking.

[29] Bai S., Zi Y. (2004). Vitamins in steel—Rare earth metals. Met. World.

[30] Chen Y. (2005). Promising application of rare earths in steel and non-ferrous metals. Rare Earth Inf..

[31] Xu G. (1995). Rare Earths.

[32] Cao X. (2013). Effect of Rare Earth Metal Cerium on the Texture and Properties of X80 Pipeline Steel. Master’s Thesis.

[33] Yu Z. (1984). Application of Rare Earths in Steel.

[34] Wang F. (2010). Influence of Y-mg Alloy on Structure and Properties in SS400 Steel. Master’s Thesis.

[35] Waudby P. (1978). Rare earth additions to steel. Int. Met. Rev..

[36] Lin Q., Ye W., Yu Z. (1990). Behavior of rare earths in solid solution in steel. Chin. J. Met. Sci. Technol..

[37] Du T., Wang L. (1985). Thermodynamics of Fe-Y-S, Fe-Y-O and Fe-Y-S-O metallic solutions. J. Less Common Met..

[38] Wu Y., Wang L., Du T. (1985). Thermodynamics of rare earth elements in liquid iron. J. Less Common Met..

[39] Pang F., Yin S., Li J., Wang S. (2011). Study on deoxidization thermodynamics of trace rare earth element and formation mechanism of inclusions in LZ50 molten steel. J. Taiyuan Univ. Technol..

[40] Zheng Z., Chen K., Suo J., Chen F. (2001). Effect of rare earth element on desulphurization of steel in continuous casting. Spec. Steel.

[41] Ma J., Liu F. (2009). Application of rare earth element in steel and its influence on steel properties. Res. Iron Steel.

[42] Zhou L., Tang L., Miao D., Sun W., Huang H. (2001). Rare earth alloying and its effect on law of precipitation of V and Nb precipitated phase. Res. Iron Steel.

[43] Ji J., Che Y., Liu A., Lu X., You M. (1996). Research and theory in internal friction of alloying of rare earth in iron and steel. J. Chin. Rare Earth Soc..

[44] Jin Z. (1997). A study on rare earth metal microalloying of alloy steel. Spec. Steel.

[45] Wang X., Hao K., Zhou G., Wu H., Wu R. (2012). Effect of rare-earth on sulfides morphology and abrasive resistance of high sulfur steel. Mater. Mech. Eng..

[46] Mei Z., Wan T., Lou D. (2002). Influence of RE modifier on as-cast grain refinement of super-low carbon cast steel. Spec. Cast. Nonferrous Alloy..

[47] Liu C., Fang L., Huang G., Wang Y., Chen J., Li C., Jiang M. (2008). Microstructure of high -carbon RE steel. Chin. Rare Earths.

[48] Zhang H., Zhao L., Qin X., Sun X. (2013). Effect of cerium on microstructure and hardness of 00Cr17Mo stainless steel. Chin. Rare Earths.

[49] Lan J., He J., Ding W., Wang Q., Zhu Y. (2000). Effect of Rare Earth Metals on the Microstructure and Impact Toughness of a Cast 0.4 C–5Cr–1.2 Mo–1.0 V Steel. ISIJ Int..

[50] Fu H., Xiao Q., Kuang J., Jiang Z., Xing J.-D. (2007). Effect of rare earth and titanium additions on the microstructures and properties of low carbon Fe–B cast steel. Mater. Sci. Eng. A.

[51] Samanta S., Mitra S., Pal T. (2006). Effect of rare earth elements on microstructure and oxidation behaviour in TIG weldments of AISI 316L stainless steel. Mater. Sci. Eng. A.

[52] Kesri R., Durand-Charre M. (1987). Phase equilibria, solidification and solid-state transformations of white cast irons containing niobium. J. Mater. Sci..

[53] Ramar A., Spätig P., Schäublin R. (2008). Analysis of high temperature deformation mechanism in ODS EUROFER97 alloy. J. Nucl. Mater..

[54] Capdevila C., Chen Y., Lassen N.K., Jones A., Bhadeshia H. (2001). Heterogeneous deformation and recrystallisation of iron base oxide dispersion strengthened PM2000 alloy. Mater. Sci. Technol..

[55] Malik M.A., Us Salam I., Muhammad W., Ejaz N. (2009). Effect of microstructural anisotropy on mechanical behavior of a high-strength Al–Mg–Si Alloy. J. Fail. Anal. Prev..

[56] Humphreys F., Ardakani M. (1996). Grain boundary migration and Zener pinning in particle-containing copper crystals. Acta Mater..

[57] Bate P. (2001). The effect of deformation on grain growth in Zener pinned systems. Acta Mater..

[58] Chen L., Ma X., Wang L., Ye X. (2011). Effect of rare earth element yttrium addition on microstructures and properties of a 21Cr–11Ni austenitic heat-resistant stainless steel. Mater. Des..

[59] Rios P.R., Siciliano F., Sandim H.R.Z., Plaut R.L., Padilha A.F. (2005). Nucleation and growth during recrystallization. Mater. Res..

[60] Andersen I., Grong Ø., Ryum N. (1995). Analytical modelling of grain growth in metals and alloys in the presence of growing and dissolving precipitates—II. Abnormal grain growth. Acta Metall. Mater..

[61] Hotta S., Yamada K., Murakami T., Narushima T., Iguchi Y., Ouchi C. (2006). *β* Grain Refinement due to Small Amounts of Yttrium Addition in. *α* + *β* Type Titanium Alloy, SP-700. ISIJ Int..

[62] Yang J., Zou D., Li X., Du Z. (2007). Effect of rare earth on microstructures and properties of high speed steel with high carbon content. J. Iron Steel Res. Int..

[63] Xu Z. (2000). Influence of Ce and Al on nodularization of eutectic in austenite–bainite steel. Mater. Res. Bull..

[64] Qian M. (1999). *In situ* observations of the dissolution of carbides in an Fe-Cr-C alloy. Scr. Mater..

[65] Wang S., Dai T. (1986). Relaxational Internal Friction Peak of in a Co-base Superalloy. Acta Metall. Sin..

[66] Yang Q.-X., Liao B., Liu J., Yao M. (1998). Effect of rare earth elements on carbide morphology and phase transformation dynamics of high Ni-Cr alloy cast iron. J. Rare Earths.

[67] Chen X., Li Y. (2007). Fracture toughness improvement of austempered high silicon steel by titanium, vanadium and rare earth elements modification. Mater. Sci. Eng. A.

[68] Edmonds D., Cochrane R. (1990). Structure-property relationships in bainitic steels. Metall. Trans. A.

[69] Lan J., He J., Ding W., Wang Q., Zhu Y. (2001). Study on heterogeneous nuclei in cast H13 steel modified by rare earth. J. Rare Earths.

[70] Bhadeshia H., Edmonds D. (1983). Bainite in silicon steels: New composition–property approach Part 1. Met. Sci..

[71] Bhadeshia H., Edmonds D. (1983). Bainite in silicon steels: New composition–property approach Part 2. Met. Sci..

[72] Miihkinen V., Edmonds D. (1987). Fracture toughness of two experimental high-strength bainitic low-alloy steels containing silicon. Mater. Sci. Technol..

[73] Miihkinen V., Edmonds D. (1987). Tensile deformation of two experimental high-strength bainitic low-alloy steels containing silicon. Mater. Sci. Technol..

[74] Miihkinen V., Edmonds D. (1987). Microstructural examination of two experimental high-strength bainitic low-alloy steels containing silicon. Mater. Sci. Technol..

[75] Wang M., Mu S., Sun F., Wang Y. (2007). Influence of rare earth elements on microstructure and mechanical properties of cast high-speed steel rolls. J. Rare Earths.

[76] Liu R., Yan M., Wu D. (2010). Microstructure and mechanical properties of 17-4PH steel plasma nitrocarburized with and without rare earths addition. J. Mater. Process. Technol..

[77] Yan M., Sun Y., Bell T., Liu Z., Xia L. (2000). Diffusion of La in plasma RE ion nitrided surface layer and its effect in nitrogen concentration profiles and phase structures. Acta Metall. Sin..

[78] Yan M. (2002). Effect of temperature and phase constitution on kinetics of La diffusion. J. Rare Earths.

[79] Park S., Jung I., Oh K., Lee H. (2004). Effect of Al on the evolution of non-metallic inclusions in the Mn-Si-Ti-Mg deoxidized steel during solidification: Experiments and thermodynamic calculations. ISIJ Int..

